# Specific Antecedents of Entrepreneurial Intention Among Newly Returned Chinese International Students

**DOI:** 10.3389/fpsyg.2021.622276

**Published:** 2021-04-22

**Authors:** Yue Mao, Yinghua Ye

**Affiliations:** ^1^Department of Educational and Counselling Psychology, and Special Education, Faculty of Education, The University of British Columbia, Vancouver, BC, Canada; ^2^College of Education, Zhejiang University, Hangzhou, China

**Keywords:** entrepreneurial intention, newly returned Chinese international students, bicultural identity integration, ambidextrous social network, bilingual proficiency, entrepreneurship education

## Abstract

A growing group of Chinese students is returning to China following graduation, especially young returnees. This group is seen as one of the most innovative sectors of Chinese society. Based on the theory of planned behavior (TPB) and three kinds of capital theories, this study explores entrepreneurial intention (EI) and its influencing factors among Newly Returned Chinese International Students (NRCIS). A survey of 211 NRCIS showed a low level of EI and little knowledge of supporting policies about entrepreneurship. Influencing factors included culture harmony as culture capital, overseas social networks as social capital, and foreign entrepreneurship education and foreign language proficiency as human capital. Attitude mediated the effects of foreign language proficiency, culture harmony, and foreign entrepreneurship education on EI. Perceived behavior control mediated the effect of foreign language proficiency, Chinese language proficiency, culture harmony, foreign entrepreneurship education, domestic entrepreneurship education, and overseas social networks on EI, and subjective norms have no significant mediating effect in any mediation path. Based on these findings, policymakers could pay attention to examining whether the current policies are working and accessible for NRCIS, and domestic entrepreneurship education could keep cultivating students' cross-cultural communication and understanding abilities, and society and education sectors could encourage positive cognition of entrepreneurship and guide students to form a positive attitude toward entrepreneurship and enhance their confidence.

## Introduction

For decades, Chinese students have been going abroad for their education, and many of them choose to stay and pursue careers overseas. However, with the rapid development of China's social economy, a growing number of returnees has been a decade-long trend. According to the National Bureau of Statistics of China, the ratio of the number of students returned back over that of who went abroad in the corresponding year has increased, from 47.34% in 2010 to 82.49% in 2019.

**Figure 1:**
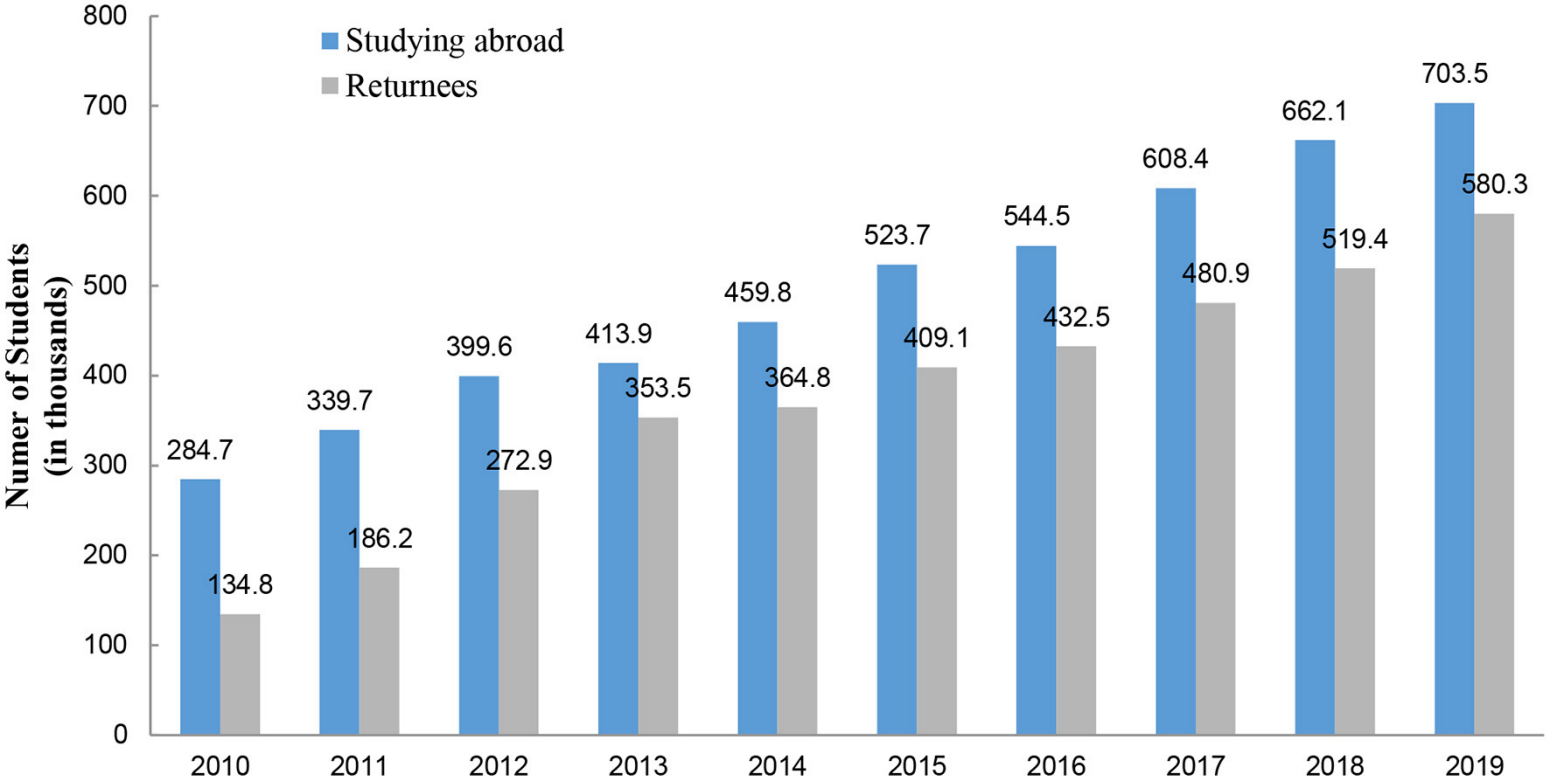
Number of Chinese students studying abroad and returned. Source: Ministry of Education of the People's Republic of China.

Figure 1 shows this increasing trend in the last 10 years. Moreover, it is noteworthy that the number of young returnees accounts for a significant proportion of returnees' total number. According to the *Report on Employment and Entrepreneurship of Chinese Returnees* (the Center for China and Globalization, 2018), more than 80% of the returnees were born between 1985 and 1995.

A series of published works discussed the trend in the context of brain gain, brain exchange, and brain circulation, as opposed to brain drain (Saxenian, 2005; Docquier and Rapoport, 2012; Giannetti et al., 2015). The Chinese government initiated support policies to encourage venture creation to unleash returnees' potential (Wang and Liu, 2016; Xia et al., 2020). However, despite the favorable policy toward returnee entrepreneurs, a survey showed that 95.2% of the young returnees in Hunan province did not enjoy any support policies for their employment and entrepreneurship, and 80.9% of the young returnees with entrepreneurial experience are funded by relatives and friends (Zhong, 2016).

Therefore, the target population of the present study is the NRCIS, specifically those who were born after 1980, underwent public or self-funded study at a formal overseas university or academic institution and successfully graduated with a bachelor's or higher degree, and have received degree certification from the Overseas Education Service Center of the Ministry of Education. They usually return to China within 3 years after graduation. It is reasonable to look at the EI and the potential predictors of this young, vital, and yet neglected group. The research questions are as follows:

1. What is their level of EI?
2. What factors affect their EI, especially factors that may be specific to them? For example, does the entrepreneurship education they received at home and abroad influence their EI in the same way?

This study contributes to the EI literature by investigating the EI of an interesting and enlarging yet uninvestigated group of people. Moreover, it can be used to improve practices in policymaking and entrepreneurship education.

The following section introduces the theory of planned behavior (TPB), a frequently used model to research EI, and three kinds of capital theories, the framework we use to analyze the characteristics of the NRCIS and find potential predictors of their EI. Next, the paper presents the study's theoretical framework and hypotheses, followed by the materials and methods used to measure the variables and collected data. In the data analysis process, we used structural equation modeling to test the hypotheses. Finally, we discuss the results and propose some implications.

### Entrepreneurial Intention and the TPB

#### Entrepreneurial Intention (EI)

EI is the belief in planning entrepreneurial action in the future (Tsai et al., 2016). It provides a way to explain and predict entrepreneurship and has been attracting researchers' attention for decades (Krueger et al., 2000). There are numerous studies on EI that explore its predictors, which can be divided into individual internal factors such as personality (Brandstätter, 2011; Mathieu and St-Jean, 2013), and external environmental factors such as social environment (Santos et al., 2016), and some demographic factors (Díaz-García and Jiménez-Moreno, 2010). Studies also analyzed the EI of certain groups, such as university students, middle school students, farmers, and migrant workers. Predictors of EI are different across these groups; for example, entrepreneurship education is an essential predictor for students (Zhang et al., 2014) but probably not of the same significance for farmers (Khoshmaram et al., 2020) and migrant workers (Duan et al., 2020). There are few studies on EI and the predictors of NRCIS. A survey-based on 288 returnees showed that the education degree earned overseas, the business model brought from overseas, and whether the business was in the secondary industry had a significant and positive relationship with returnees EI (Miao et al., 2015). Fu and Si (2018) studied the effect of second-generation returnees on family firm corporate entrepreneurship in China and found a positive relationship. The longer they stay overseas, the more likely they are to promote corporate entrepreneurship. Our study attempted to enrich the literature of NRCIS' EI and evaluate what factors contribute to their EI, including factors commonly shared by other groups and those specific for young international graduates. We first focused on the framework of TPB.

#### TPB

Among studies on EI, the TPB has been widely practiced (Gird and Bagraim, 2008; Maresch et al., 2016; Miranda et al., 2017). Developed from the theory of rational action, TPB holds that human behavior is the result of planning. The intention is the predictive variable of behavior, and attitude (A, refers to one's own positive or negative feelings about an act/ entrepreneurship), subjective norms (SN, refers to one's perception of others' social pressure for or against an act/entrepreneurial behaviors), and perceived behavior control (PBC, refers to individuals' cognition of the available resources and foreseeable obstacles for an act/ a start-up business) are three antecedents of intention, with other individual and environmental factors working by influencing these three factors (Ajzen, 1991, 2011). Given the pervasive application of TPB in EI research (Souitaris et al., 2007; Díaz-García and Jiménez-Moreno, 2010; Liñán et al., 2011), an empirical study used longitudinal data to investigate the effectiveness of TPB in predicting EI and subsequent entrepreneurial behavior, and the results support a model showing that A, SN, and PBC are significant predictors for EI. Subsequently, EI and PBC significantly predict entrepreneurial behavior (Kautonen et al., 2013).

Some previous studies assume that A, SN, and PBC mediate other predictors' effects on EI. Peng et al. (2012) have explored the mediating effect of entrepreneurial attitudes, subjective norm, and entrepreneurial self-efficacy between the relationship of gender, prior entrepreneurial experience, personality traits, and others, and EI of university students. Moreover, researchers have tested the mediating effect of personal attitude, perceived social norms, and perceived feasibility between the relationship of entrepreneurial knowledge and EI (Liñán et al., 2011).

Based on the literature mentioned above, the current study explores A, SN, and PBC's mediating role between the different kinds of capital and EI of NRCIS. The next section provides a brief overview of three different kinds of capital and those predictors.

### Capital Theories and Characteristics of Returned International Students

Three kinds of capital theories analyze capital at the individual level, including the cultural, social, and human capital theories of representative scholars such as Coleman, Bourdieu, and Becker. Social capital, acquisition, and the utilization of social network resources emphasize the “mutual recognition and acknowledgment” of individuals through “investment in social networks”; cultural capital is the “reproduction of dominant values” which focuses on the “internalization or misrecognition of dominant values”; human capital is the “accumulation of surplus value through investment in skills and knowledge” (Lin, 1999).

Three kinds of capital theories provide perspectives to depict the characteristics of returned international students. Given that these returnees have studied in foreign countries for a considerable time, they are likely to be influenced by both the mainstream culture of their country of origin and host countries (Chen et al., 2008), which can be seen as the cultural capital. They also develop a social network in the two countries, which can be seen as social capital (Wahba and Zenou, 2012). These students primarily gain knowledge and skills through overseas education, which can be seen as human capital (Liu et al., 2010). Such capital can be useful for venture creation and potentially influence young returnees' EI.

#### Bicultural Identity Integration (BII)

Some refer to what we term the cultural capital of international students as biculturalism possessed by people who have been exposed to a second culture for a long time. To quantify the returned international students' attributes, we chose the variable BII, which captures the degree to which people view their dual cultural identities as compatible. High BII individuals can shift appropriately between cultures and respond in a culturally congruent way (Benet-Martínez et al., 2002). No study has explored the direct relationship between BII and EI. This study considers the merit gained from staying overseas as being that it improves the usage of cultural knowledge, psychological adjustment, sociocognitive skills, and creativity (Tadmor et al., 2009; Mok and Morris, 2010; Chen et al., 2013). These subsequently affect the returned international students' EI.

#### Ambidextrous Social Network (ASN)

The current study considers returnees' social capital mainly as social network resources both locally and overseas. According to Pruthi (2014), local ties and local networking are indispensable for returnee entrepreneurs to start a venture. Furthermore, overseas networks are seen as a unique advantage for returnee entrepreneurs (Qin and Estrin, 2015). ASN is a network that has both close local connections and international connections. Returnees are relatively likely to have family members, friends, classmates, tutors, and colleagues, both in their country of birth and abroad, compared with other groups. It can be a unique strength when engaged in entrepreneurial activities. Therefore, the present study includes ASN, which consists of local social networks (LSN) and overseas social networks (OSN), as a potential predictor of EI among NRCIS.

#### Bilingual Proficiency (BP) and Entrepreneurship Education (EE)

As for human capital, it is an increment of knowledge and skill. The overseas education experience can lead to an increase in knowledge and skills in many aspects. Considering the impact on EI, we chose BP and EE as potential predictors.

**BP** is proficiency in two languages: the ability and skill to use them competently. In the current research context, one of the languages is Chinese, and the other language can be any language used abroad. It is a common belief that exposure to a second language is of great use in picking up a second language. Young returnees have experienced living in environments where they use one or both languages daily. They may have better language abilities. As a cross-cultural communication tool, language ability is considered necessary for start-ups (Johnstone et al., 2018). It is reasonable that BP may increase young returnees' confidence in entrepreneurial activity. Therefore, this study examines the effect of BP on EI.

**EE** consists of programs, courses, workshops, contests, and other content organized by universities to help students build the skills to identify opportunities and encourage entrepreneurial activity, such as creating a new product or service, opening a company, or helping charitable organizations. Numerous universities across nations provide EE (Robinson and Haynes, 1991; Iacobucci and Micozzi, 2012; Semenov and Eremeeva, 2016), and reports often show how successful EE is in creating entrepreneurial climates, increasing entrepreneurial intentions, and building student entrepreneurs (Raposo and do Paço, 2011; Zhang et al., 2014; Maresch et al., 2016; Bergmann et al., 2018; Ni and Ye, 2018). Therefore, we examined the effect of EE on the EI of returnees. Because EE has been a global trend for decades (Kuratko, 2005), both EE received in the home country and abroad are considered.

In summary, overseas study backgrounds bring NRCIS unique cultural, social, and human capital. These capitals may have an impact on their EI. Specifically, in this study, BII represents cultural capital, ASN represents social capital, BP and EE represent students' human capital.

### Theoretical Model and Hypotheses

**Figure 2:**
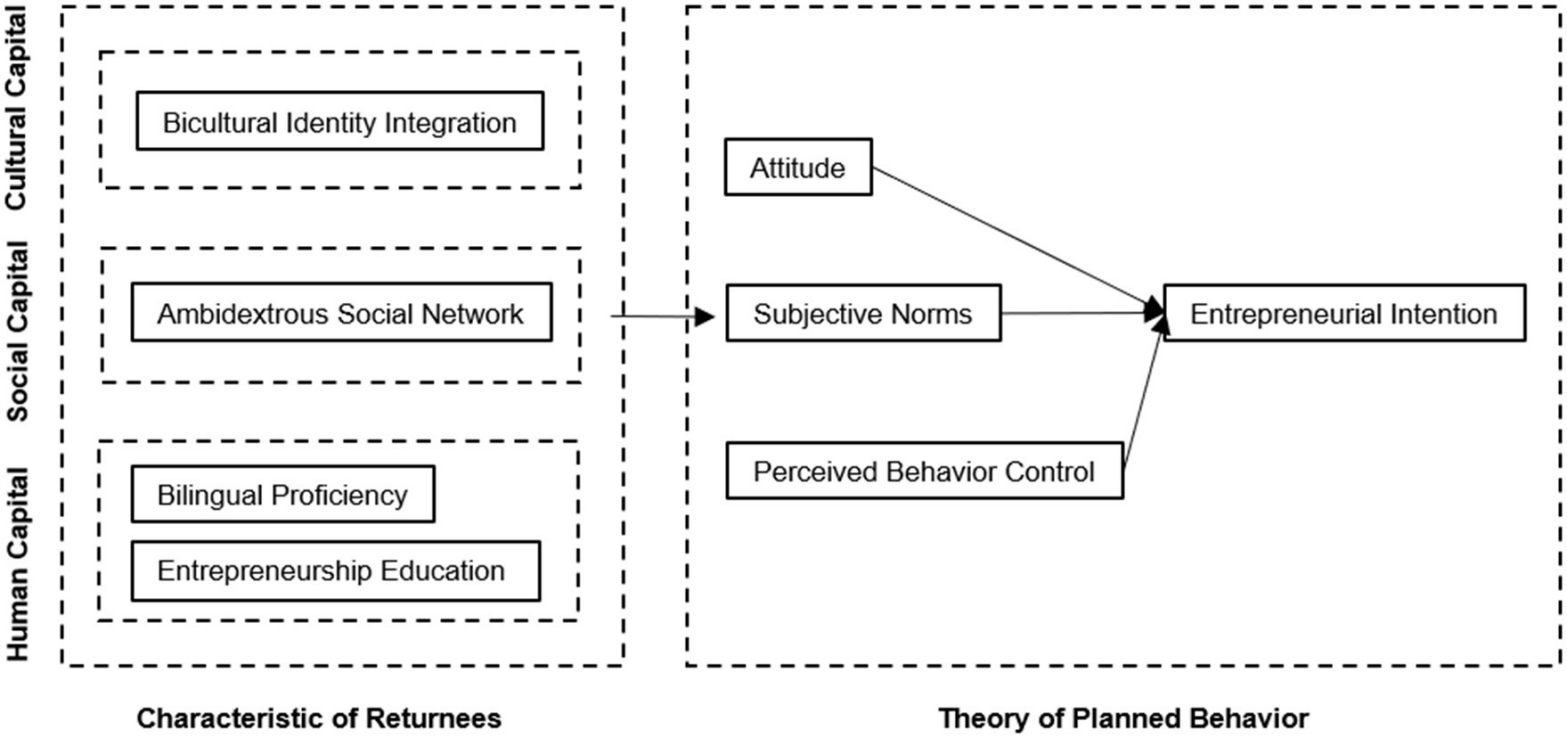
Theoretical framework.

The following theoretical framework (Figure 2) summarizes the research design; the study focuses on the following issues:

(a) What is the level of EI of NRCIS, and are there demographic differences?
(b) Do BII, ASN, BP, and EE influence their EI?
(c) Are A, SN, and PBC mediators of the relationship between predictors and EI in (b)?

The main hypotheses are (1) all the potential predictors listed here have a significant effect on EI, and (2) these predictors affect EI through A, SN, and PBC.

## Materials and Methods

### Sample and Procedure

This study employed an online questionnaire. Because of the difficulty in recruiting large-scale participants of the target population, we adopted snowball sampling. Samples were taken by recommendation and re-recommendation, which is possible when samples are difficult to obtain (Goodman, 1961). The initial samples include friends of the researchers' acquaintances, and the following samples include friends of the initial samples.

From December 2018 to March 2019, the researchers collected 255 questionnaires. Among them, 211 were valid, making for an effective rate of 82.75%. The sample size of 211 is acceptable for the current study as it is more than the minimum sample size suggested by the rule of thumb, that is, at least 100 samples (Boomsma, 1985) and 10 cases per variable (Nunnally, 1967). Table 1 describes the characteristics of the sample. One hundred sixty-nine participants were born in the 1990's, with the rest being born in the 1980's. The number of females (114) is slightly greater than that of males (97). Fourteen participants had entrepreneurial experience, 95 thought of it, and 102 had never considered entrepreneurial behavior. A large percentage of the participants (137) had little knowledge of the supporting policies of returnee entrepreneurship, and only 14 reported an awareness of the relevant policies. According to their knowledge of the policies, several participants chose not to respond to the reasonability (43) and accessibility (47) of these policies. Those who responded generally tended to think that policy reasonability was average or above but that policy accessibility was average or below.

**Table 1:** Descriptive statistics of the sample.

**Demographic variables**	**Level**	**Frequency**	**Percentage**
Year of birth	1980–1989	42	19.91
	1990–1999	169	80.09
Gender	Male	97	45.97
	Female	114	54.03
Family residence	City	144	68.25
	Town	43	20.38
	Countryside	24	11.37
Siblings	Zero	148	70.14
	At least one	63	29.86
Degree	BA	45	21.33
	MA	132	62.56
	PhD	34	16.11
Starting of oversea	High school	12	5.69
education	Undergraduate	65	30.81
	Master	116	54.98
	Doctor	18	8.53
Destination of oversea	Asia	30	14.22
study	Europe	91	43.13
	North America	73	34.60
	Australia	17	8.06
Major	Natural science	25	11.85
	Medical and pharmaceutical science	4	1.90
	Engineering and technology	62	29.38
	Humanities and social sciences	119	56.40
	Missing	1	0.47
Oversea intern	Yes	74	35.07
experience	No	137	64.93
Oversea work	Yes	26	12.32
experience	No	185	87.68
Entrepreneurial	Yes	78	36.97
experience of Close family members (CFM)	No	133	63.03
Entrepreneurial	Have never thought of	102	48.34
experience of the participant	Have thought of but have not taken action	84	39.81
	Have not taken action but with plan	11	5.21
	Currently in practice	8	3.79
	Used to but not currently in practice	6	2.84
Knowledge of the	Quite lack of	77	36.49
supporting policies (SP)	Somewhat lack of	60	28.44
	Average	60	28.44
	Somewhat know	12	5.69
	Quite know	2	0.95
Perceived reasonability	Quite unreasonable	2	0.95
of the supporting policies	Somewhat unreasonable	5	2.37
	Average	118	55.92
	Somewhat reasonable	42	19.91
	Quite reasonable	1	0.47
	Missing	43	20.38
Perceived accessibility	Quite inaccessible	9	4.27
of the supporting	Somewhat inaccessible	44	20.85
policies	Average	81	38.39
	Somewhat accessible	29	13.74
	Quite accessible	1	0.47
	Missing	47	22.27
Total		211	100

### Measures

#### Outcome Variable

##### EI

The six-item EI scale developed by Liñán and Chen (2009) was translated into Chinese and cut to four items for brevity. The remaining items, such as “I have very seriously thought about starting a firm someday.” “The scale was given in seven points, with “1” representing “totally disagree” and “7” representing “totally agree.” Confirmatory factor analysis showed good structural validity. The fit indices of the unidimensional structure were χ^2^/*df* = 1.983, GFI = 0.991, AGFI = 0.954, NFI = 0.994, IFI = 0.997, and RMSEA = 0.068.

#### Predictors

##### BII

This study used BIIS-2 (Huynh, 2009) to measure BII. For questionnaire brevity, only six items (cultural harmony and cultural distance dimension, CH & CD) were adapted and retained, such as “I find it easy to balance both Chinese culture and the culture of the other country (cultural harmony dimension” and “I know the differences between the two cultures clearly (cultural distance dimension). “The answers were on a 5-point rating scale ranging from” “1” (“totally disagree”) to “5” (“totally agree”). Confirmatory factor analysis showed good structural validity. The fit indices of the two-dimensional structure were χ^2^/*df* = 1.773, GFI = 0.980, AGFI = 0.946, NFI = 0.969, IFI = 0.986, and RMSEA = 0.061.

##### ASN

This study adapted the ASN scale from Yuan and Xiao (2013), which contained 12 items for local and overseas social networks; the local network includes the local business network (LBN), which includes connection with local industry alliances, sellers and other companies, and the local institutional network (LIN), includes connections with local government, venture capital companies, and financial institutions. The overseas network includes the overseas market network (OMN), which includes connections with overseas business partners, suppliers, and customers, and the overseas technique network (OTN), which includes connections with overseas research institutes, universities, and academic staff. The answers were on a 5-point rating scale, with “1” representing that the respondent has no such connection, and “5,” indicating that the respondent has many such connections. Confirmatory factor analysis showed good structural validity. The fit indices of the four-dimensional structure were χ^2^/*df* = 2.568, GFI = 0.909, AGFI = 0.851, NFI = 0.946, IFI = 0.966, and RMSEA = 0.086.

##### BP

We measured BP using an adapted version of the Foreign and Chinese language proficiency and usage scale (FLP & CLP) developed by Benet-Martínez and Haritatos (2005). The adapted scale had eight items measuring the use-frequency of the foreign language used in the other country and Chinese in childhood and adulthood as well as when communicating with friends and reading newspapers and magazines. The answers were on a 6-point rating scale with “1” representing “almost never” and “6” representing “very often.” Confirmatory factor analysis showed good structural validity. The fit indices of the two-dimensional structure were χ^2^/*df* = 1.289, GFI = 0.973, AGFI = 0.948, NFI = 0.957, IFI = 0.990, and RMSEA = 0.037.

##### EE

We measured EE using the scale used by Xu et al. (2016). It contains eight items asking respondents whether they attended any course on economics, entrepreneurial theory, entrepreneurial awareness, or entrepreneurial practice in China or other countries (domestic and foreign EE, DEE & FEE). The items were answered on a 3-point rating scale where “1” represents “no,” “2” represents “unclear,” and “3” represents “yes.” Confirmatory factor analysis showed good structural validity. The fit indices of the two-dimensional structure were χ^2^/*df* = 2.539, GFI = 0.948, AGFI = 0.901, NFI = 0.945, IFI = 0.966, and RMSEA = 0.086.

#### Mediators

##### A, SN, and PBC

The scale developed by Liñán and Chen (2009), with 14 items measuring potential mediators, was taken from TPB. A includes five items (e.g., “Being an entrepreneur would entail great satisfaction for me”), SN includes three (e.g., “If you decided to create a firm, would your close family approve of that decision?”). Furthermore, PBC includes six (e.g., “I can control the creation process of a new firm”). The answers were given on a 7-point rating scale ranging from 1 (totally disagree) to 7 (totally agree). Confirmatory factor analysis showed good structural validity. The fit indices were χ^2^/*df* = 3.073, GFI = 0.855, AGFI = 0.794, NFI = 0.910, IFI = 0.938, and RMSEA = 0.099.

#### Cronbach's Alpha, Composite Reliability (CR) and Average Variance Extracted (AVE) of Each Research Variables

According to Nunnally (1967) and Fornell and Larcker (1981), when the Cronbach's Alpha> 0.7, CR > 0.7, AVE > 0.5, it indicates that the consistency between the items is acceptable (Yang, 2016). When the CR > 0.7 and AVE > 0.5, the convergence validity of the variable is high (Yang, 2016). As shown in Table 2, the variables in the present study have good reliability and convergence validity.

**Table 2:** Cronbach's alpha, CR, and AVE of each research variables (*N* = 211).

	**EI**	**BII**		**ASN**				**BP**		**EE**		**TPB**		
		**CH**	**CD**	**LBN**	**LIN**	**OMN**	**OTN**	**FLP**	**CLP**	**DEE**	**FEE**	**A**	**SN**	**PBC**
Alpha	0.93	0.82	0.79	0.88	0.88	0.90	0.91	0.81	0.79	0.81	0.84	0.93	0.83	0.94
CR	0.93	0.82	0.79	0.88	0.89	0.91	0.92	0.81	0.81	0.84	0.85	0.93	0.85	0.94
AVE	078	0.61	0.57	0.71	0.72	0.76	0.78	0.52	0.52	0.58	0.61	0.72	0.66	0.72

### Testing of Common Method Biases

This study mainly uses the self-report scale to collect data, and the same data collection methods may produce common method biases (Tang and Wen, 2020). Therefore, Harman's single-factor test was used to test the common method bias. Results of exploratory factor analysis showed that a total of 12 factors were exacted with eigenvalues >1 when unrotated, and the amount of variance explained by the first common factor was 25.79%, which was less than the critical value of 40%. Therefore, the current study reported no serious standard method bias.

### Data Analysis

This study used SPSS20.0 and AMOS21.0 for data processing. First, we used SPSS to run the descriptive statistical analysis and test the significance of each variable's demographic differences. Second, we checked the correlation between these variables. Finally, we used AMOS and Bootstrap methods to conduct structural equation modeling (SEM) to further explore the mediating effect.

## Results

### Descriptive and Correlational Results

Table 3 shows the descriptive and correlational results. As Table 3 shows, NRCIS reported low scores of EI (mean = 3.70, 7-point) and PBC (mean = 3.12, 7-point), although they held a positive A (mean = 4.60, 7-point) and SN (mean = 4.97, 7-point) toward entrepreneurship. The correlational results show that EI had significant positive correlations with almost all the variables.

**Table 3:** Mean, SD and correlations (*N* = 211).

	**M**	**SD**	**1**	**2**	**3**	**4**	**5**	**6**	**7**	**8**
1. BII	3.64	0.65	1							
2. ASN	3.58	0.84	0.33^**^	1						
3. BP	4.44	0.72	0.38^**^	0.28^**^	1					
4. EE	1.88	0.60	0.16^*^	0.09	0.10	1				
5. A	4.60	1.40	0.20^**^	0.28^**^	0.14^*^	0.28^**^	1			
6. SN	4.97	1.25	0.32^**^	0.32^**^	0.30^**^	0.16^*^	0.56^**^	1		
7. PBC	3.12	1.43	0.27^**^	0.21^**^	0.13	0.46^**^	0.58^**^	0.45^**^	1	
8. EI	3.70	1.67	0.27^**^	0.27^**^	0.19^**^	0.38^**^	0.74^**^	0.51^**^	0.75^**^	1

* *p < 0.05*.

** *p < 0.01*.

### Demographic Differences in Each Variable

Independent *t*-test and one-way ANOVA were employed to find differences in the main variables at different levels of the demographic variables, such as gender and education (Table 4).

**Table 4:** Demographic differences in the scores for main variables.

	**Gender**	**Degree**	**Major**	**E-experience of CFM**	**E-experience of the participant**	**Knowledge of the SP**
BII	Male > female^*^	No difference	No difference	Y > N^*^	No difference	No difference
ASN	No difference	No difference	No difference	No difference	No difference	No difference
BP	No difference	No difference	Humanities and social > medical and pharmaceutical science^*^	Y > N^*^	No difference	No difference
EE	No difference	BA > Ph.D.^*^	Other > medicaland pharmaceutical science^*^	No difference	No difference	Quite lack of < average^***^
A	Male > female^*^	No difference	No difference	Y > N^*^	“Have never thought of” had the lowest scores	“Quite lack of” had the lowest scores
SN	Male > female^*^	No difference	No difference	Y > N^**^	“Have never thought of” had the lowest scores	“Quite lack of” had the lowest scores
PBC	Male > female^**^	BA > MA**, Ph.D.^**^	No difference	Y > N^**^	“Have never thought of” had the lowest scores	“Quite lack of” had the lowest scores
EI	Male > female^*^	BA > Ph.D.^*^	No difference	Y > N^***^	“Have never thought of” had the lowest scores	“Quite lack of” had the lowest scores

* *p < 0.05*.

** *p < 0.01*.

*** *p < 0.001*.

NRCIS with more entrepreneurial experiences, more knowledge of related supporting policies, and close family members with entrepreneurial experiences scored higher on EI as well as in A, SN, and PBC. Male participants scored higher in BII, EI, A, SN, and PBC. Participants with a bachelor's degree had more EE and PBC and higher EI than those with a master's or doctoral degree. Participants born in the 1990's reported more entrepreneurship education than those born in the 1980's. Participants who started overseas education as undergraduates also reported more entrepreneurship education than those who started high school. Participants with internship experience had a more pronounced bilingual proficiency. The year of birth, the year starting overseas education, and overseas internship experience are not shown in Table 4 because they only show differences in one variable, which is not very important. Furthermore, four demographic variables, family residence, siblings, overseas study destination, and work experience are not shown in the table because they did not contribute to any differences in the key variables.

### Mediating Analysis

Casual Steps Approach and Products of Coefficients are commonly used methods in mediating analysis. The coefficient product method directly tests the significance of the product of ab, so the Non-parametric Bootstrap Method (Bias-corrected), which is a method of Product of Coefficients, was used in the current study. The mediating effect of A, SN, and PBC was tested by the structural equation model. The sample number of Bootstrap was set as 5,000, and the confidence interval (CI) was set as 95%. If 0 is excluded from the upper and lower limits of the 95% CI, the mediating effect is significant; if 0 is included, the mediating effect is not significant.

**Figure 3:**
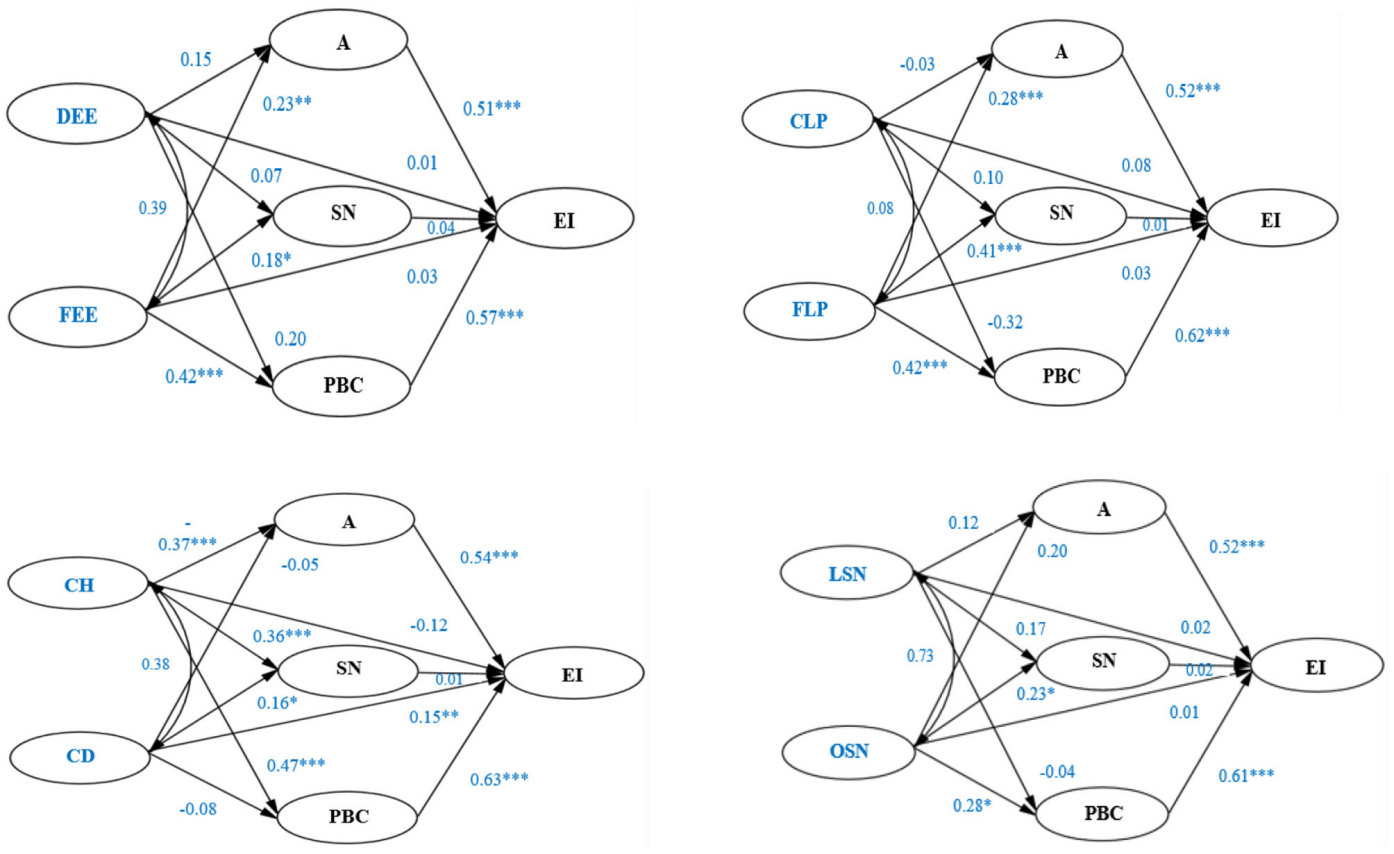
Impact of EE, BP, BII, and ASN on EI—mediated by A, SN, and PBC. *p < 0.05. **p < 0.01. ***p < 0.001.

Four mediating models were constructed to test the mediating effect of A, SN, and PBC in the process of four independent variables (EE, BP, BII, and ASN) influencing dependent variables (EI). The four models are shown in Figure 3, and the total, direct, and indirect effects of four independent variables on EI are shown in Table 5.

**Table 5:** Total, direct and indirect effect of four independent variables on EI.

**Effects**	**EE**		**BP**		**BII**		**ASN**	
	**DEE**	**FEE**	**CLP**	**FLP**	**CH**	**CD**	**LSN**	**OSN**
Total effect	0.20	0.40^***^	−0.14	0.44^**^	0.38^**^	0.07	0.06	0.29^**^
Direct effect	0.01	0.03	0.08	0.03	−0.12	0.15	0.02	0.01
Indirect effect	0.20^*^	0.37^***^	−0.21^*^	0.41^**^	0.49^**^	−0.08	0.05	0.28^*^

* *p < 0.05*.

** *p < 0.01*.

*** *p < 0.001*.

According to Figure 3 and Table 5, FEE, FLP, CH, and OSN had a significant total effect on EI, but DEE, CLP, CD, and LSN had no significant effect. Moreover, if the three mediation variables are taken as a whole, they are all significant in other paths except for the fact that the effects of CD and LSN on EI were not significantly mediated.

The estimate and significance of each mediation path are shown in Table 6. SN has no significant mediating effect in any mediation path.

**Table 6:** Estimates and significance of each mediation path.

**Mediation Path**	**Estimate**	**LLCI**	**ULCI**	***P***
**EE**				
FEE→PBC→EI	0.79	0.47	1.32	*p* < 0.001
FEE→SN→EI	0.03	−0.04	0.14	*P* > 0.05
FEE→A→EI	0.38	0.10	0.76	*p* < 0.01
DEE→PBC→EI	0.44	0.06	1.01	*p* < 0.05
DEE→SN→EI	0.01	−0.02	0.13	*P* > 0.05
DEE→A→EI	0.30	−0.03	0.77	*P* > 0.05
**BP**				
FLP→PBC→EI	0.33	0.16	0.60	*p* < 0.001
FLP→SN→EI	0.003	−0.07	0.07	*P* > 0.05
FLP→A→EI	0.18	0.05	0.39	*p* < 0.01
CLP→PBC→EI	−0.37	−0.77	−0.18	*p* < 0.001
CLP→SN→EI	0.001	−0.03	0.05	*P* > 0.05
CLP→A→EI	−0.03	−0.31	0.19	*P* > 0.05
**BII**				
CD→PBC→EI	−0.13	−1.03	0.18	*P* > 0.05
CD→SN→EI	0.002	−0.07	0.09	*P* > 0.05
CD→A→EI	−0.07	−0.89	0.26	*P* > 0.05
CH→PBC→EI	0.47	0.24	2.12	*p* < 0.01
CH→SN→EI	0.003	−0.09	0.21	*P* > 0.05
CH→A→EI	0.32	0.10	2.12	*p* < 0.01
**ASN**				
OSN→PBC→EI	0.26	0.01	0.57	*p* < 0.05
OSN→SN→EI	0.01	−0.04	0.08	*P* > 0.05
OSN→A→EI	0.16	−0.05	0.44	*P* > 0.05
LSN→PBC→EI	−0.03	−0.27	0.19	*P* > 0.05
LSN→SN→EI	0.01	−0.02	0.08	*P* > 0.05
LSN→A→EI	0.09	−0.15	0.34	*P* > 0.05

The fit indices of four mediating models are shown in Table 7. According to Table 7, all the four mediating models are acceptable.

**Table 7:** The fit dices of four mediating models.

**Model**	**χ2/df**	**IFI**	**CFI**	**RMSEA**
EE→A, SN, PBC→EI	2.48	0.91	0.90	0.08
BP→A, SN, PBC→EI	2.47	0.90	0.90	0.08
BII→A, SN, PBC→EI	2.61	0.90	0.90	0.09
ASN→A, SN, PBC→EI	2.70	0.89	0.89	0.09

## Discussion and Implications

### Low Level of EI and Little Knowledge of Supporting Policies

The mean EI score in our sample was low (3.70, <4 on a seven-point scale). In contrast, Xu et al. (2016) and Ni and Ye (2018) investigated the EI of 1,034 and 730 secondary school students using the same scale and found higher scores (3.75 and 4.69), which, from their perspective, were lower than the EI of Chinese undergraduates. It is a rough comparison that requires more rigorous research to confirm whether the difference is statistically significant. Further, more efforts are needed, at least as much as the effort needed for other groups, if Chinese society wants more entrepreneurs to show up among NRCIS. These young returnees showed little knowledge of the supportive policies for entrepreneurship specially made for them by the Chinese government. Those who know some of the supportive policies reported the low accessibility of the concrete support measure. Policymakers and practitioners could consider how to make policies known to the public or target group.

### Three Capital Theories as a Framework

The study found that the three kinds of capital theories are suitable theoretical bases for analyzing returned international students' characteristics. Guided by the three capitals, we specified four variables as potential predictors of EI among the target population, and the FEE, FLP, CH and OSN showed significant total effects on EI.

As for cultural capital, one dimension of BII, CH had a significant total effect on EI, although the other dimension, CD, did not. This result suggests that the more those returnees are attuned to Chinese culture and the culture abroad, the higher their EI will be. In this study, the BII measures young returnees' cultural capital due to their overseas study backgrounds. It describes the subjective feeling of compatibility or contradiction between the two cultures that they are experiencing. Existing research lacks literature on exploring and testing the relationship between being bicultural and EI from cultural identity integration. Based on a large number of studies on the benefits of being bicultural and multicultural (Benet-Martínez et al., 2002; Chen et al., 2008; Leung et al., 2008; Tadmor et al., 2009), this work proposed a positive correlation between the BII and EI of returnees, which is supported by empirical evidence.

As for social capital, the OSN of ASN had a significant effect on EI. However, the influence of LSN on EI is not significant. Previous studies have found that local ties and networking are essential for returnees to create a venture (Pruthi, 2014), and OSN is a unique advantage for returnees (Qin and Estrin, 2015). Our results proved that the impact of OSN is more significant for NRCIS than LSN. The relationship between ASN and EI is an exciting topic and worthy of further investigation.

In terms of human capital, it is unsurprising that FLP and FEE showed significant effects on EI. There are a few revelations according to these results. The positive influence of FEE confirmed the effectiveness of overseas study of entrepreneurship on EI. The influence of BP is hardly addressed in content research on EI and language ability, a neglected part in EE. Combining the impact of BII, European communities' key competencies for lifelong learning are insightful. Among the eight key competencies identified by the EU, three are “communication in the mother tongue,” “communication in foreign languages,” and “cultural awareness and expression” (European Communities, 2007). This study's significant predictors were overseas social networking, foreign language proficiency, and foreign entrepreneurship education; these are unique predictors for NRCIS.

### TPB

The current study supported the TPB model partly. Most of the significant relationships between EI and the predictors were mediated by at least one of the three mediators proposed in TPB. To be more precise, they are mediated by A and PBC; SN has no significant mediating effect in any mediation path. The effects of A and PBC on EI were confirmed to be significant, while SN was not. These findings suggest that others' opinions and attitudes do not influence young returnees considering whether to engage in entrepreneurial activities. This conclusion is consistent with the finding of Ceresia and Mendola (2020). Some studies have shown that the three predictors of planned behavior theory have different effects on EI. Compared with subjective norms, behavioral attitude and perceived behavioral control have more significant effects on EI (Liñán et al., 2011).

Furthermore, attitude mediates the effects of FEE, CH, and FLP on EI, and PBC meditates the effects of FEE, DEE, CH, FLP, CLP, and OSN on EI. These significant mediating effects make sense as the scope of business activities has become highly connected worldwide because of globalization (Johnstone et al., 2018). Therefore, people who accept more entrepreneurship education and feel better about bicultural harmony and foreign language competence may feel more positive and confident about entrepreneurship. These results also suggest that entrepreneurship education courses and training might enable students to gain global vision and the ability to participate in international exchanges and cooperation, encouraging international entrepreneurship education, facilitating multicultural teaching resources, and recruiting teachers with an international background (Bell et al., 2004; Elenurm, 2008; Wu and Martin, 2018).

In this study, PBC mediated the effect of OSN on EI, but the LSN did not have the same effect. Compared with LSN, OSN are of more significance in predicting NRCIS' EI. As PBC reflects their confidence in entrepreneurial activities, how to help NRCIS build their OSN is a crucial aspect to consider in policymaking.

## Conclusion

The current study contributes to the literature on EI by exploring an essential yet not much-investigated group NRCIS, introducing the three kinds of capital theories framework to analyze potential predictors, and combining planned behavior theory to explore how each of the predictors takes effect. The three kinds of capital theories contribute to perfect and supplement the diversity of the three kinds of capital and different situations of different effects. For the planned behavior theory, one of the most critical theories in the study of entrepreneurial intention is to show that SN's mediating effect is still weaker than the other two variables (A and PBC) in the NRCIS. The findings of this study suggest that overseas study has an essential effect on NRCIS' EI, which can be generalized to a certain extent.

However, for the parsimony of the model, we have not take into account the possibility of the indirect effect of SN on EI, which has been found possible by previous study (Liñán and Chen, 2009). The mediation effect of SN may be insignificant because its effect on EI is mediated by A and PBC. This is one of this study's limitations. Another limitation is the compromise of convenience samples because of the great difficulty in recruiting sufficient samples. Therefore, we would be cautious in generalizing our findings to the general population. This study is more of exploratory work. The specific antecedents that we explored add new insights to explain the core TPB model's functioning, but to the best of our knowledge, there are few prior studies that link BP, BII and ASN to EI. The interesting reulsts call for more future studies on the topic. Hopefully, future research directions can extend these findings by testing the model in other countries and regions, or by replicating the same model with new samples using better sampling strategy. Moreover, the EI of individuals tends to change over time. Bernhofer and Li (2014) investigated the EI of more than 800 students in 16 Chinese universities and found that their EI was the lowest when they graduated, while after working for 5 years, starting their own business became the top choice. Gruenhagen (2020) also found that the perception of a stable institutional environment and the support's availability might have a positive effect on returnees' EI. It is a limitation that we have not taken environment and policy as critical predictive variables. As we found that NRCIS generally were not familiar with the available entrepreneurial supporting policy and a relatively low EI, we suggest getting NRCIS to know the policies and track their EI changes.

In short, this study found that (a) the EI of NRCIS was low, and (b) CH, OSN, FEE, and FLP had a total direct effect on NRCIS' EI, and (c) FEE, CH and FLP influenced EI through A, and FLP, CLP, CH, FEE, DEE, and OSN influenced EI through PBC. Based on these results, some suggestions are carefully proposed. First, policymakers could pay greater attention to examining whether the current policies are working and accessible for NRCIS, ensure that they provide tailored policies for this young group, and ensure that they can access and understand the policies. It includes conducting specific investigations before making policy and choosing propaganda channels that reach the NRCIS. Second, domestic entrepreneurship education could keep cultivating students' cross-cultural communication and understanding abilities. Possible strategies are building entrepreneurship education platforms for exchanges and cooperation with overseas universities, using entrepreneurship education textbooks and resources with multicultural characteristics, recruiting teachers with overseas backgrounds, adopting bilingual education. Third, based on SN and PBC's significant effect on EI, society and education sectors could encourage positive cognition of entrepreneurship and guide students to form a positive attitude toward entrepreneurship and enhance their confidence.

## Data Availability Statement

The raw data supporting the conclusions of this article will be made available by the authors, without undue reservation.

## Ethics Statement

Ethical review and approval was not required for the study on human participants in accordance with the local legislation and institutional requirements. Written informed consent for participation was not required for this study in accordance with the national legislation and the institutional requirements.

## Author Contributions

YM and YY contributed at all the work of the paper together, more research design work done by YY, and more essay writing work was done by YM. Both authors contributed to the article and approved the submitted version.

## Conflict of Interest

The authors declare that the research was conducted in the absence of any commercial or financial relationships that could be construed as a potential conflict of interest.
